# Supplementary material for: Inhibition of Human Platelet Aggregation and Low-Density Lipoprotein Oxidation by Premna foetida Extract and Its Major Compounds
Source: Molecules. 2019 Apr 13;24(8):1469. doi: 10.3390/molecules24081469 (PMC6514998; doi:10.3390/molecules24081469)

SUPPORTING INFORMATION

1. Figure 1. ^{1}H NMR spectrum of compound **1**
2. Figure 2. ^{13}C NMR spectrum of compound **1**
3. Figure 3. ^{1}H NMR spectrum of compound **2**
4. Figure 4. ^{13}C NMR spectrum of compound **2**
5. Figure 5. ^{1}H NMR spectrum of compound **3**
6. Figure 6. ^{13}C NMR spectrum of compound **3**
7. Figure 7. ^{1}H NMR spectrum of compound **4**
8. Figure 8. ^{13}C NMR spectrum of compound **4**
9. Figure 9. ^{1}H NMR spectrum of compound **5**
10. Figure 10. ^{13}C NMR spectrum of compound **5**
11. Figure 11. ^{1}H NMR spectrum of compound **6**
12. Figure 12. ^{1}H NMR spectrum of compound **7**
13. Figure 13. ^{13}C NMR spectrum of compound **7**
14. Figure 14. ^{1}H NMR spectrum of compound **8**
15. Figure 15. ^{13}C NMR spectrum of compound **8**
16. Figure 16. ^{1}H NMR spectrum of compound **9**
17. Figure 17. ^{13}C NMR spectrum of compound **9**
18. Figure 18. ^{1}H NMR spectrum of compound **10**
19. Figure 19. ^{13}C NMR spectrum of compound **10**
10\. Figure 20. ^{1}H NMR spectrum of compound **11**
11\. Figure 21. ^{13}C NMR spectrum of compound **11**

Figure 1. ^{1}H NMR spectrum of compound **1** (500 MHz, in DMSO-*d*)

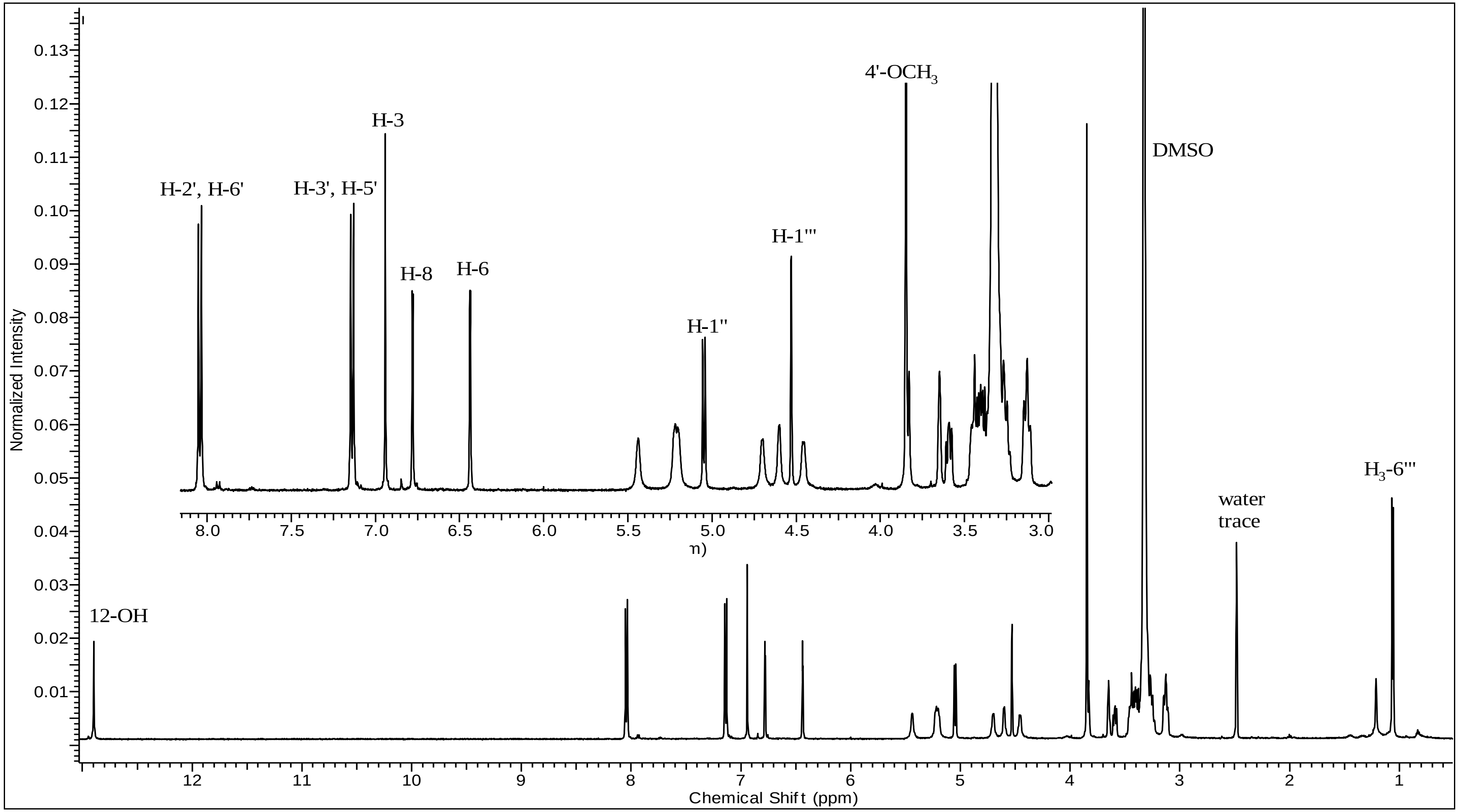

Figure 2. ^{13}C NMR spectrum of compound **1** (125 MHz in DMSO-*d*)

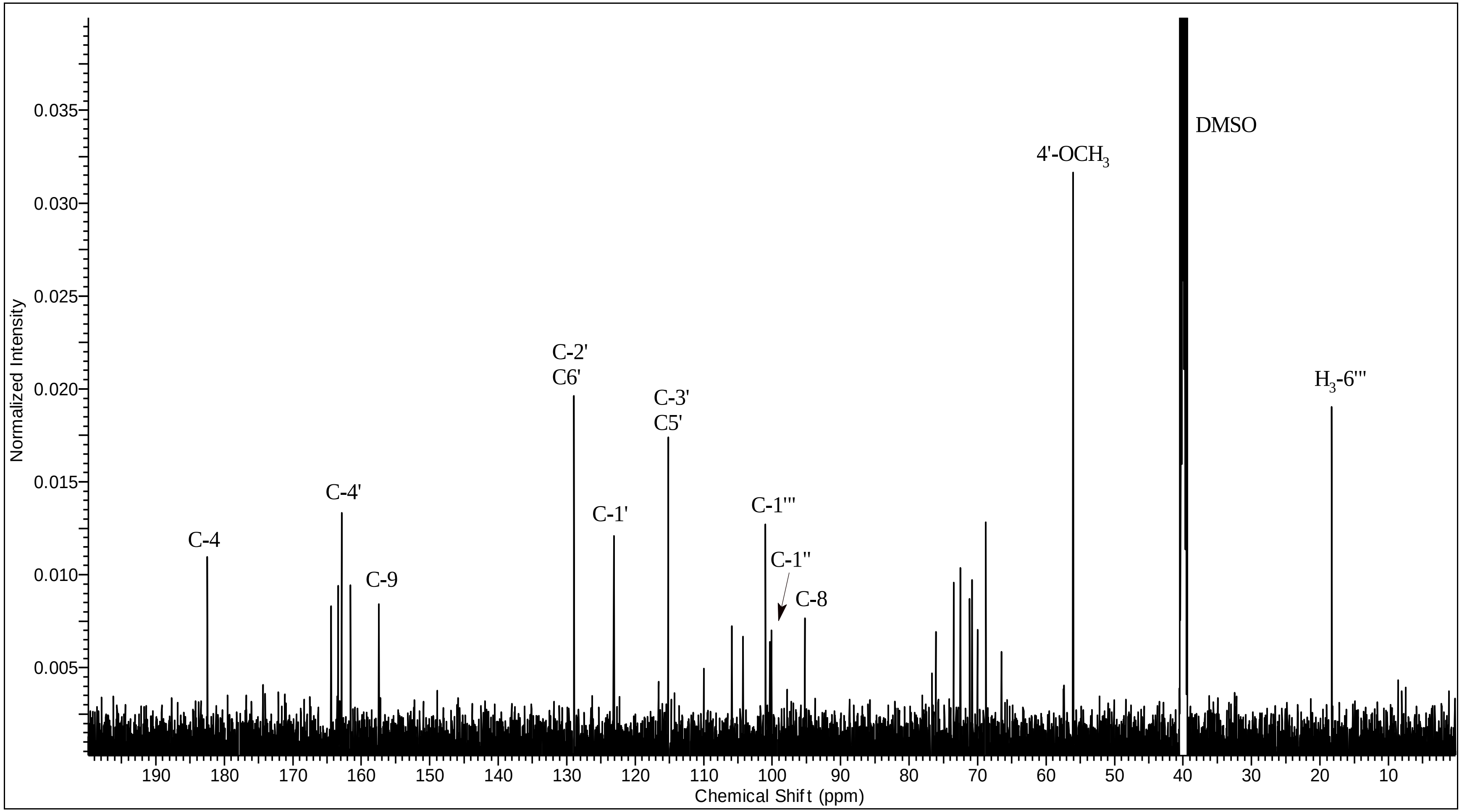

Figure 3. ^{1}H NMR spectrum of compound **2** (500 MHz in CD_3OD)

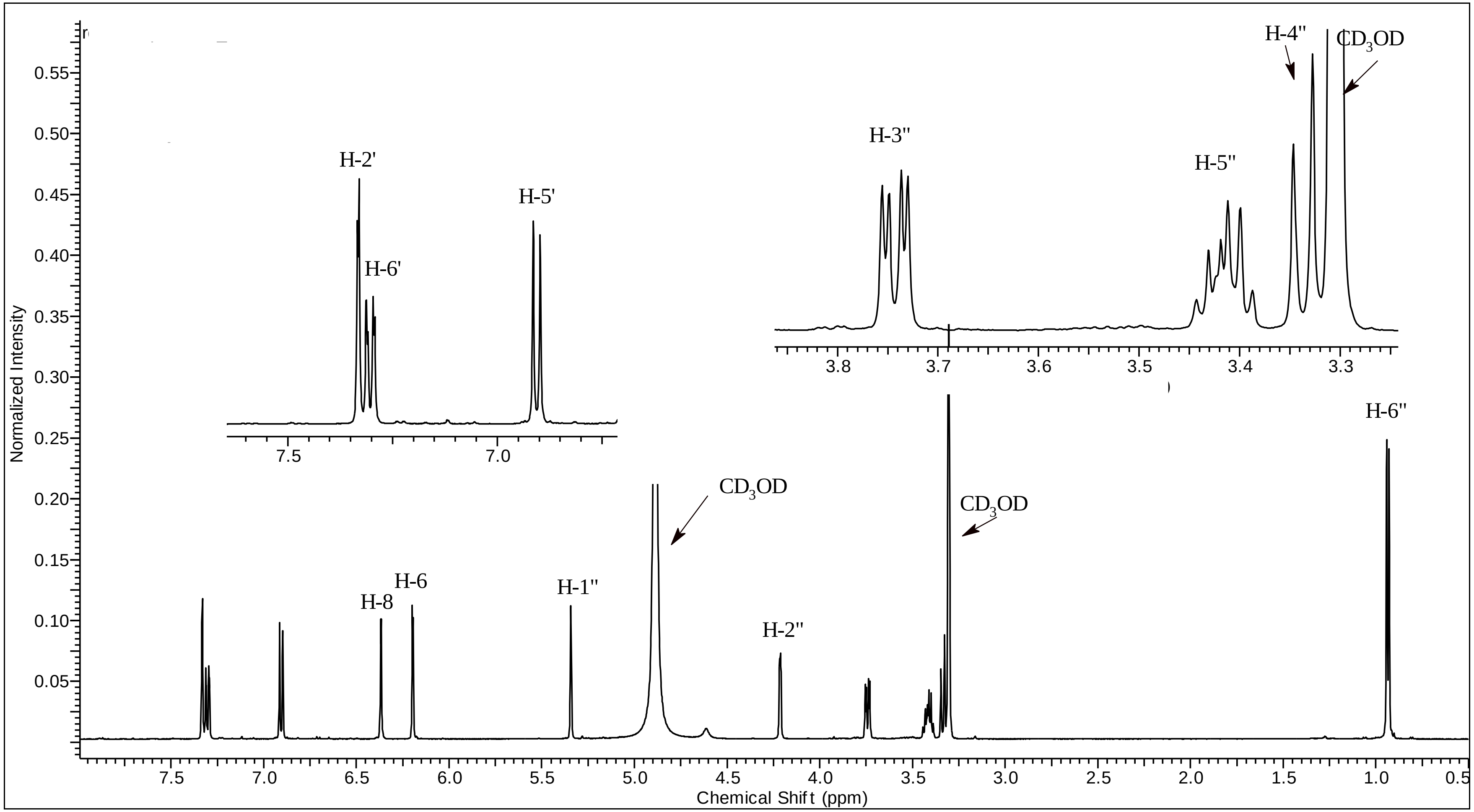

Figure 4. ^{13}C NMR spectrum of compound **2** (125 MHz in CD_3OD)

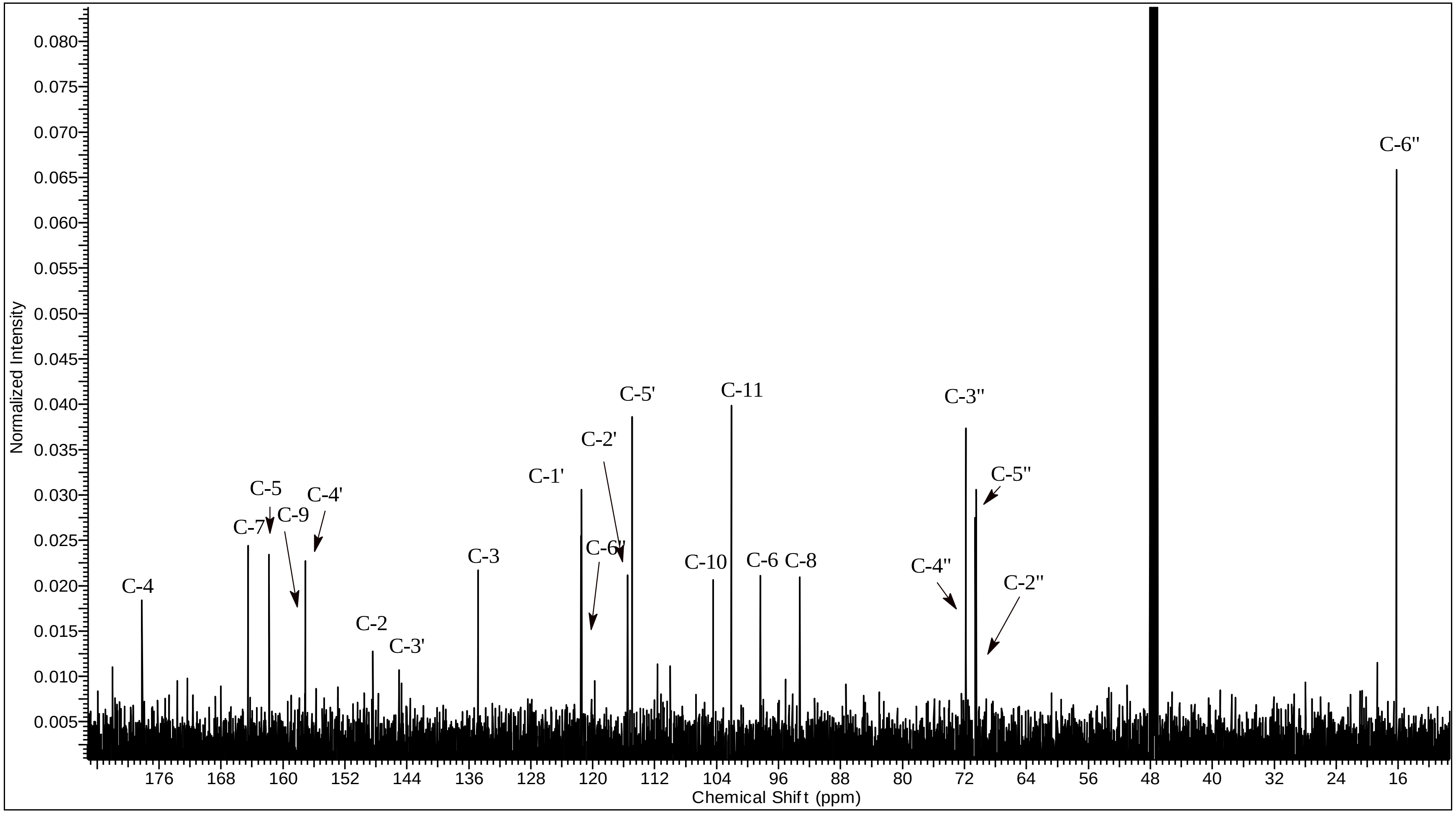

Figure 5. ^{1}H NMR spectrum of compound **3** (500 MHz in CD_3OD+$CDCl_3$)

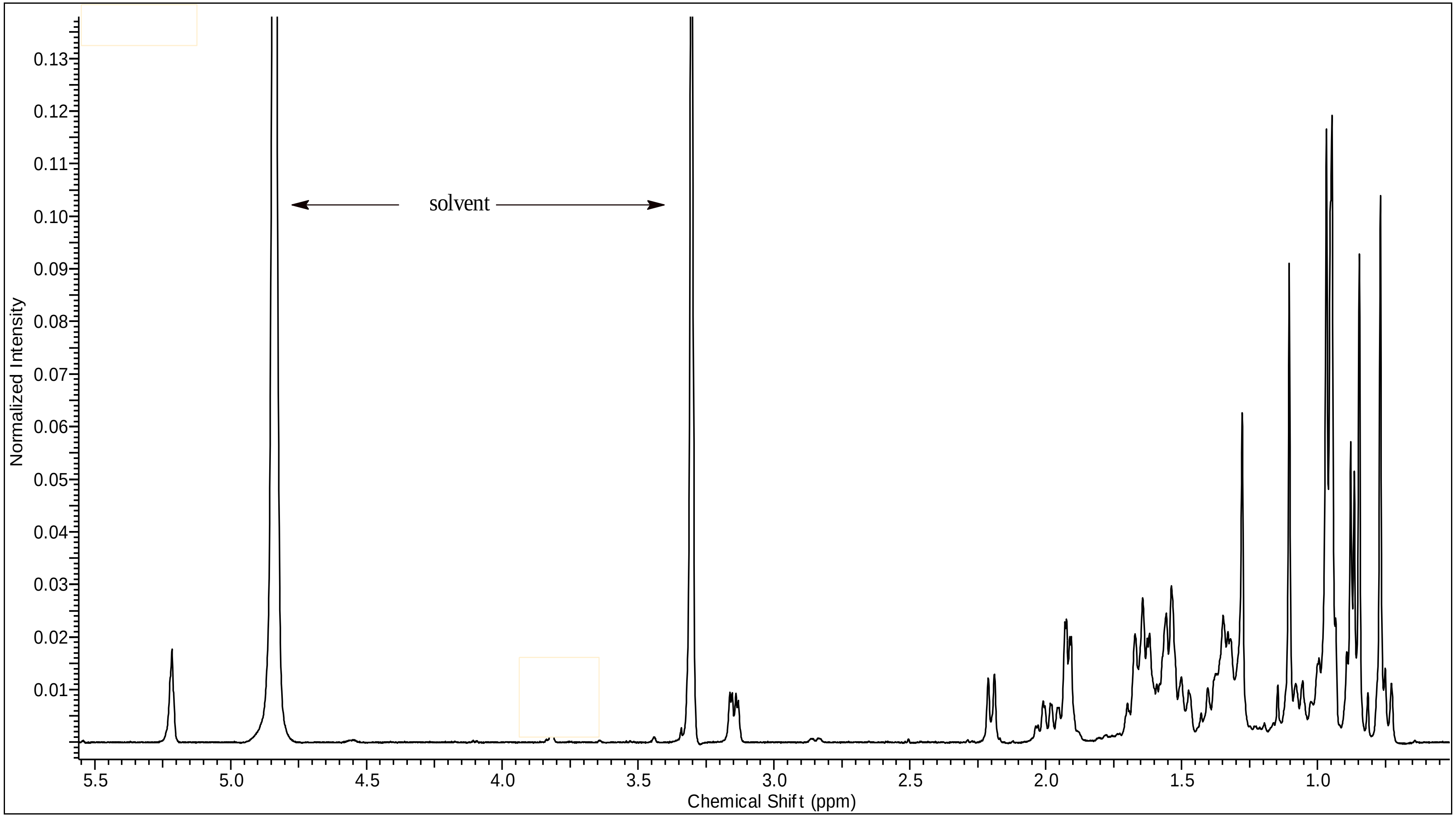

Figure 6. ^{13}C NMR spectrum of compound **3** (125 MHz in $CD_3OD+CDCl_3$)

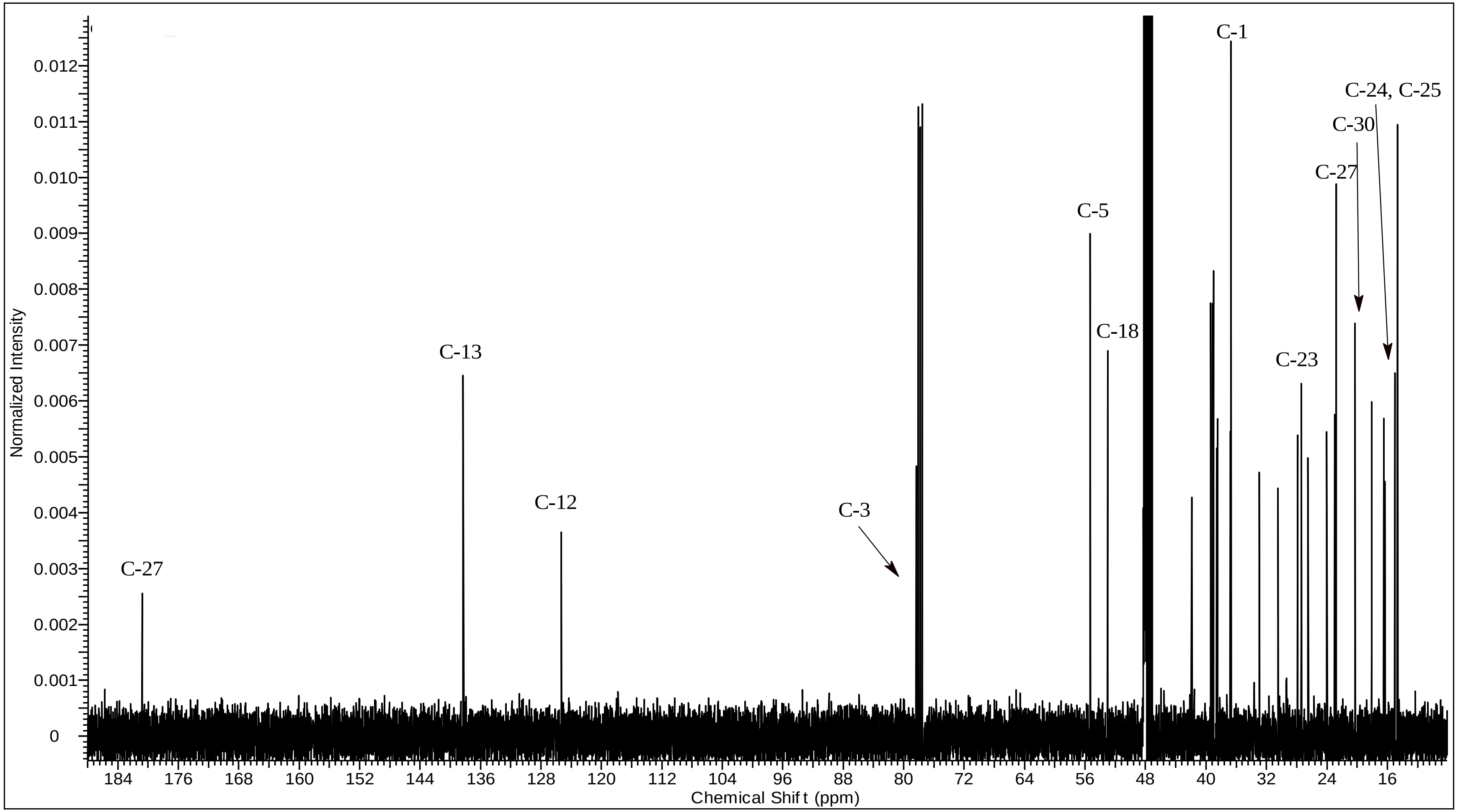

Figure 7. ^{1}H NMR spectrum of compound **4** (500 MHz in $CDCl_3$)

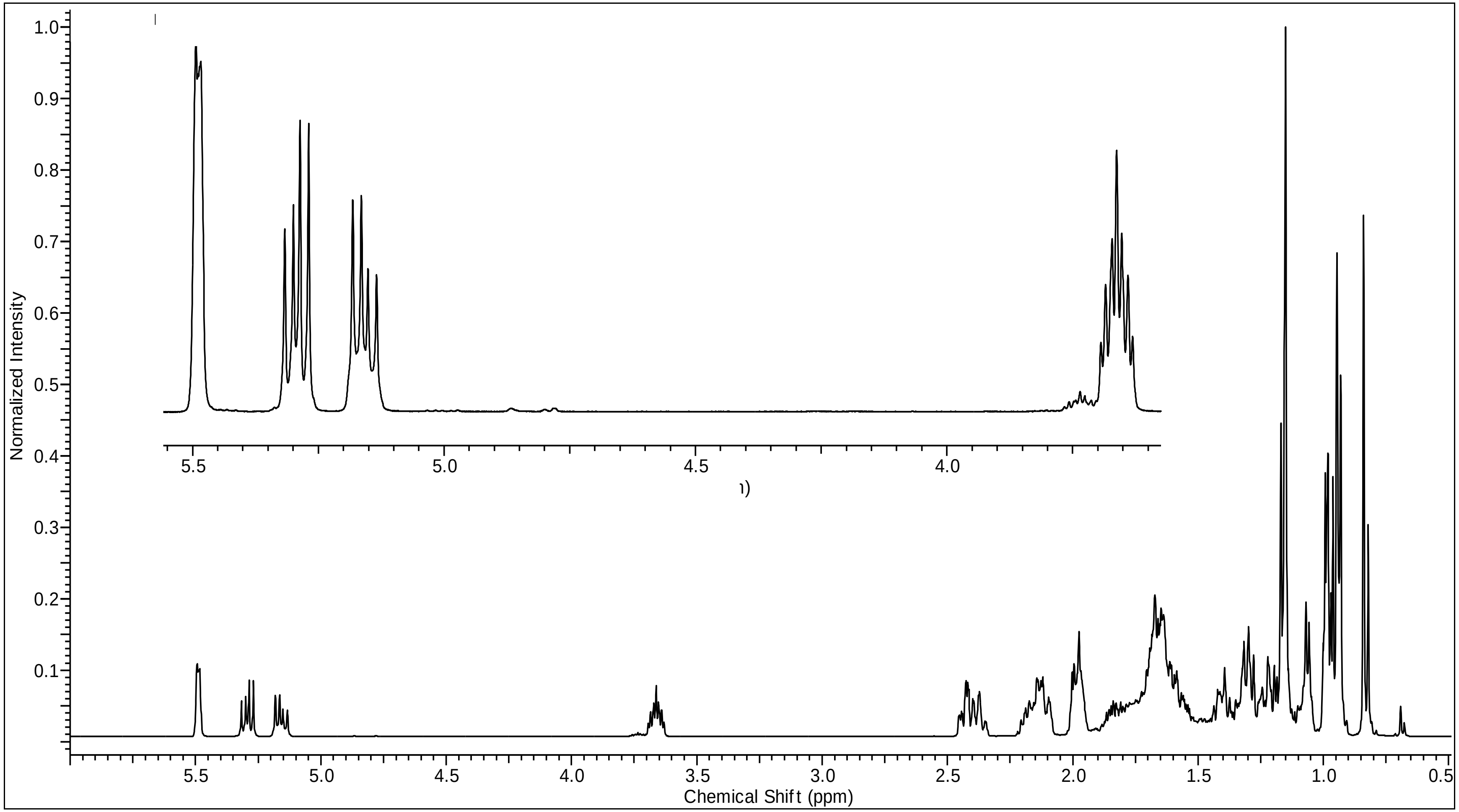

Figure 8. ^{13}C NMR spectrum of compound **4** (125 MHz in $CDCl_3$)

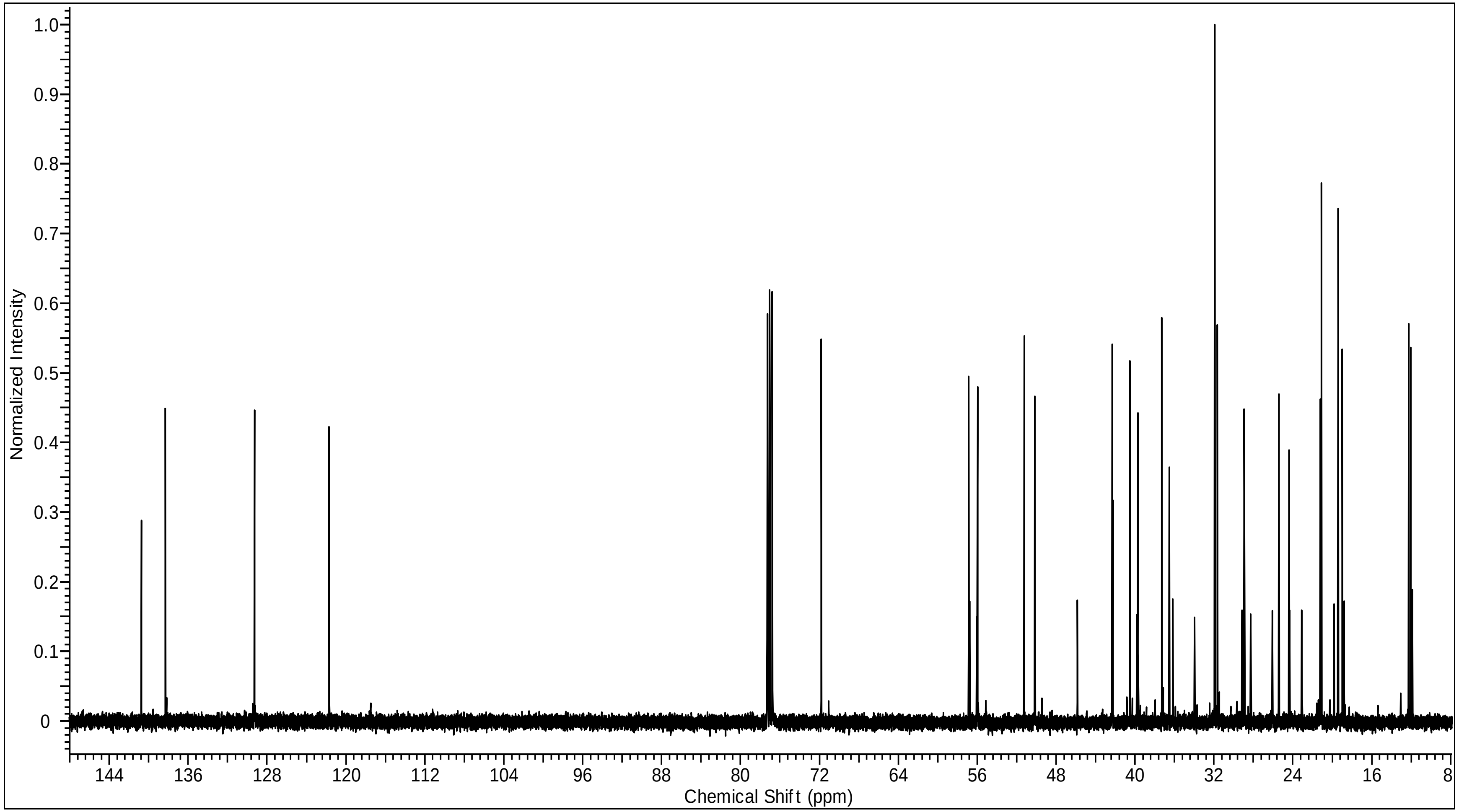

Figure 9. ^{1}H NMR spectrum of compound **5** (500 MHz in $CD_3OD+CDCl_3$)

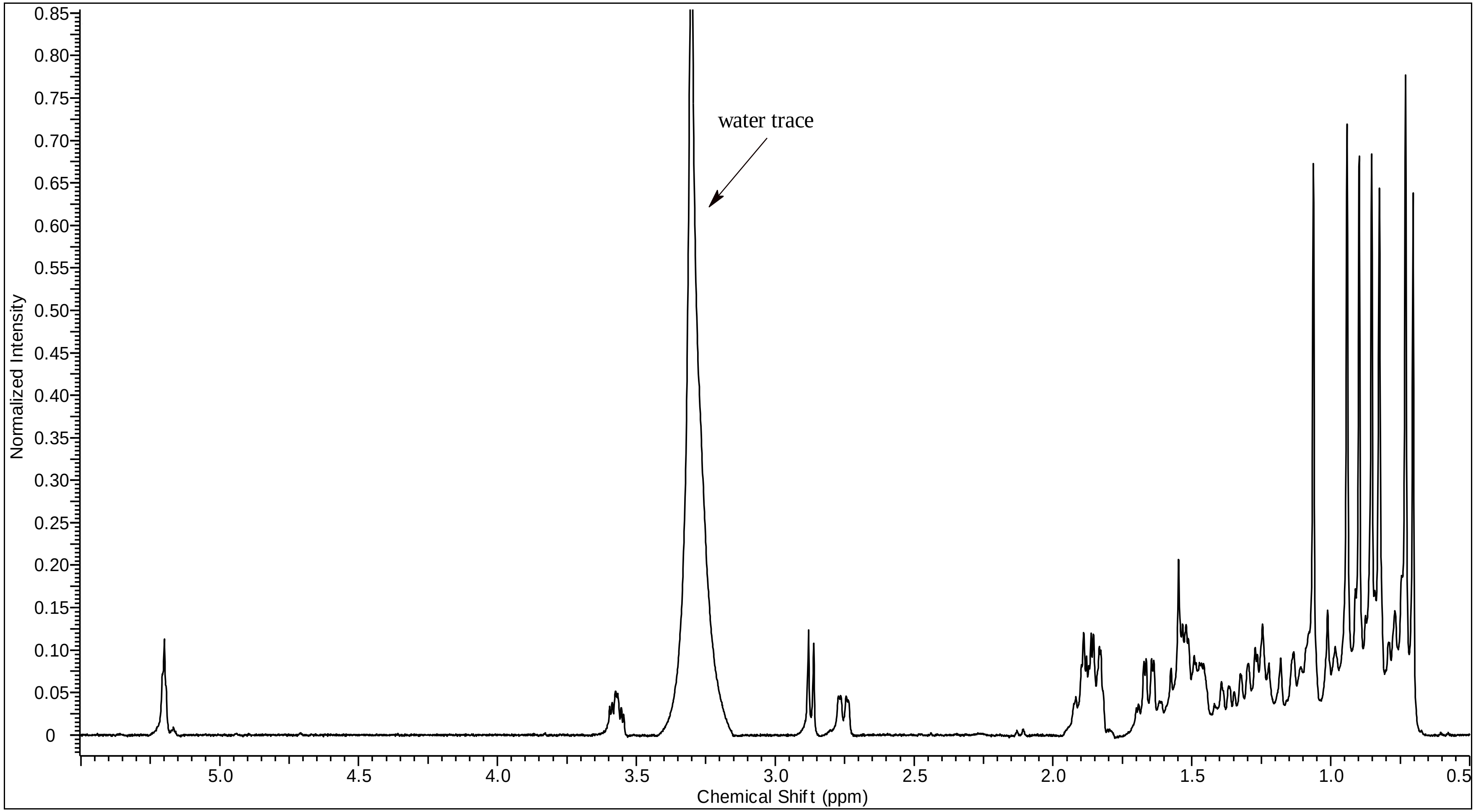

Figure 10. ^{13}C NMR spectrum of compound **5** (125 MHz in $CD_3OD+CDCl_3$)

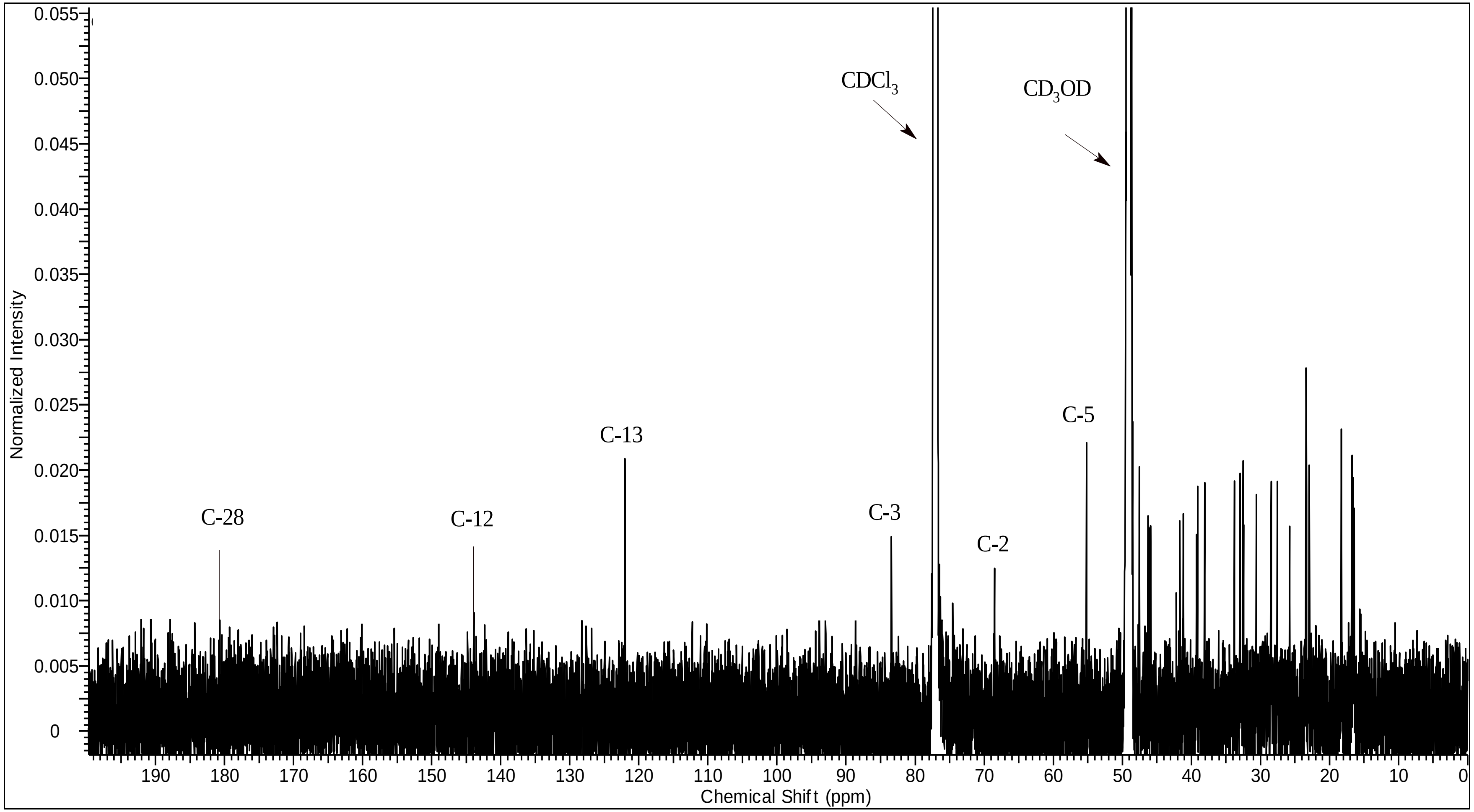

Figure 11. ^{1}H NMR spectrum of compound **6** (500 MHz in CD_3OD)

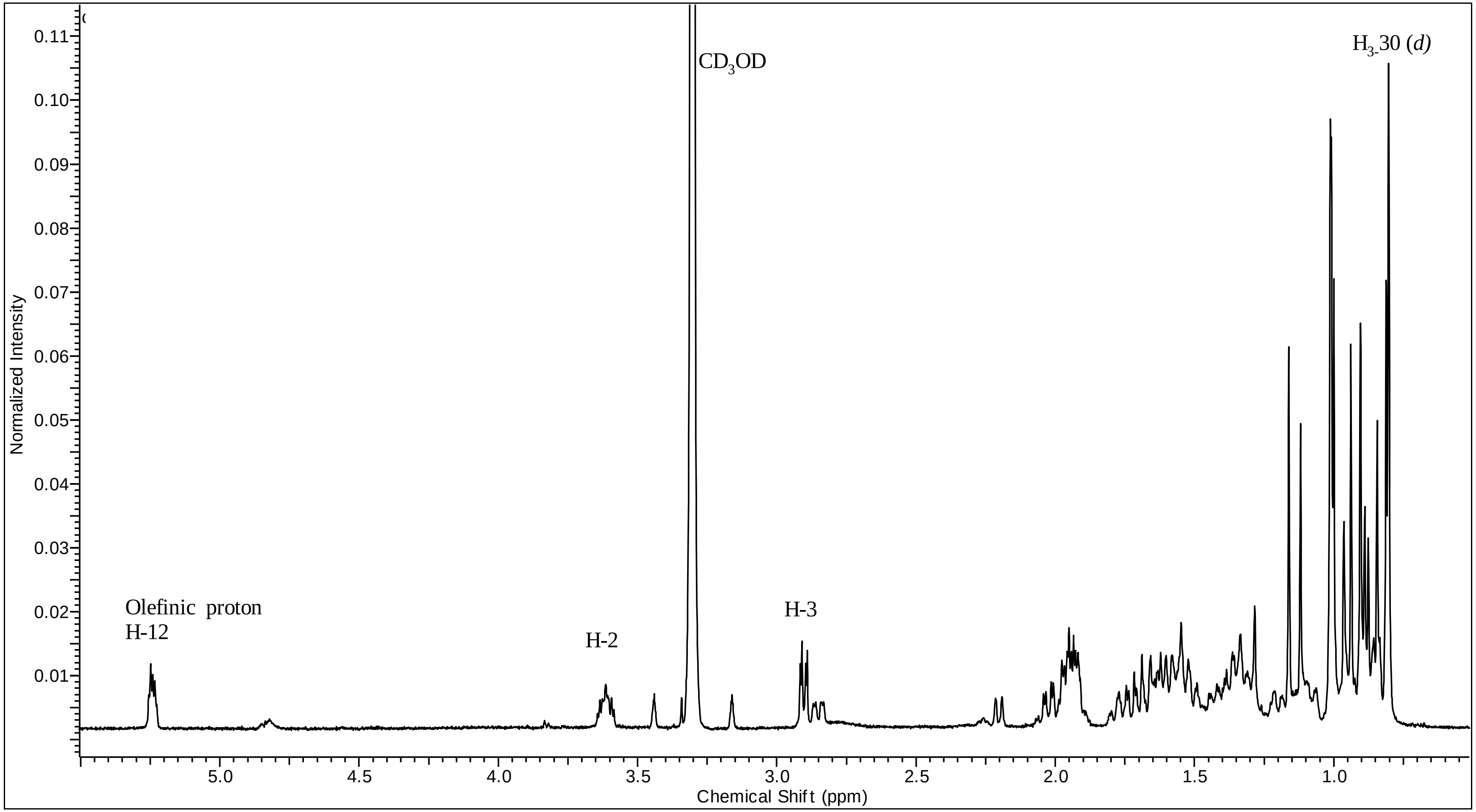

Figure 12. ^{1}H NMR spectrum of compound **7** (500 MHz in $CD_3OD+CDCl_3$)

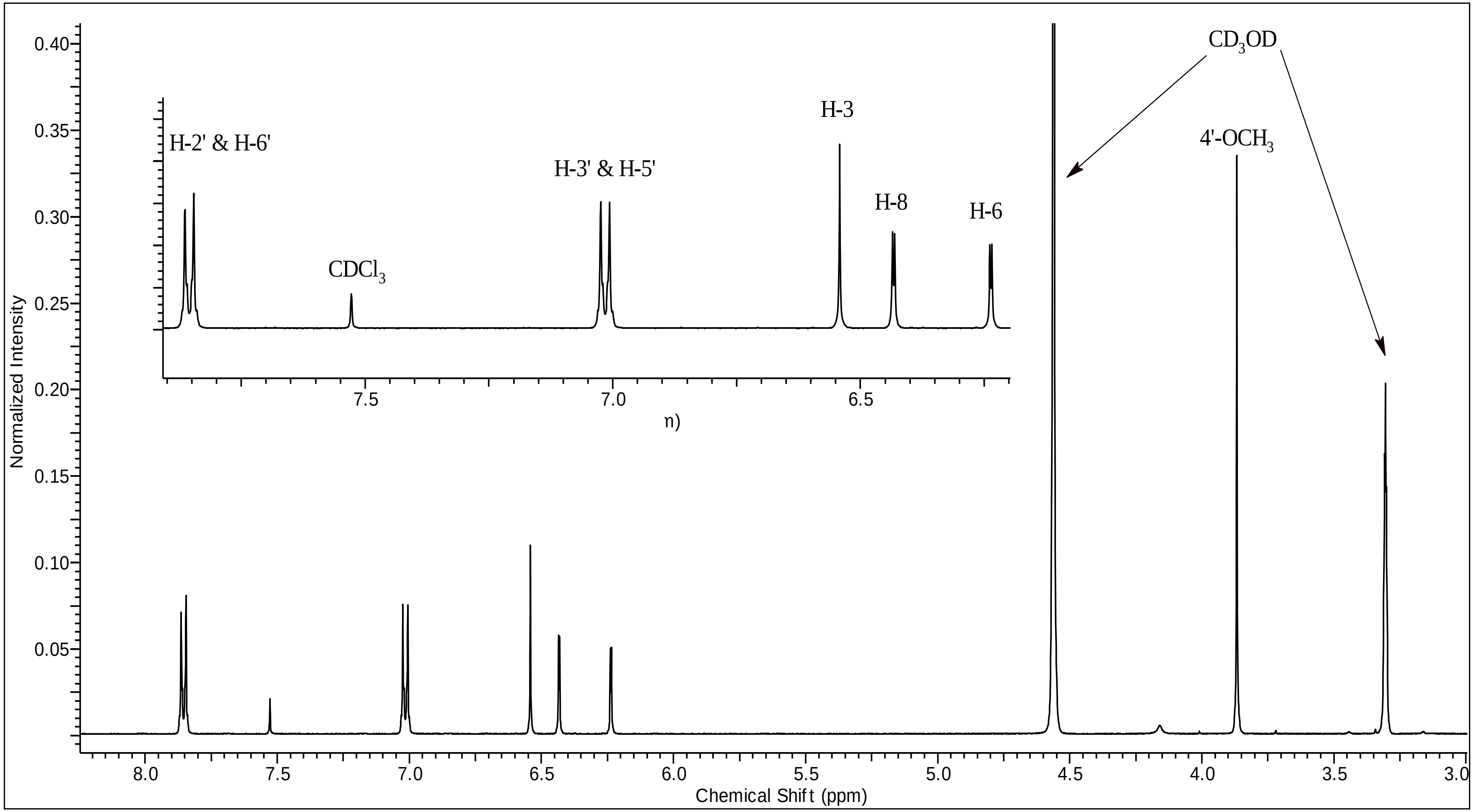

Figure 13. ^{13}C NMR spectrum of compound **7** (125 MHz in $CD_3OD+CDCl_3$)

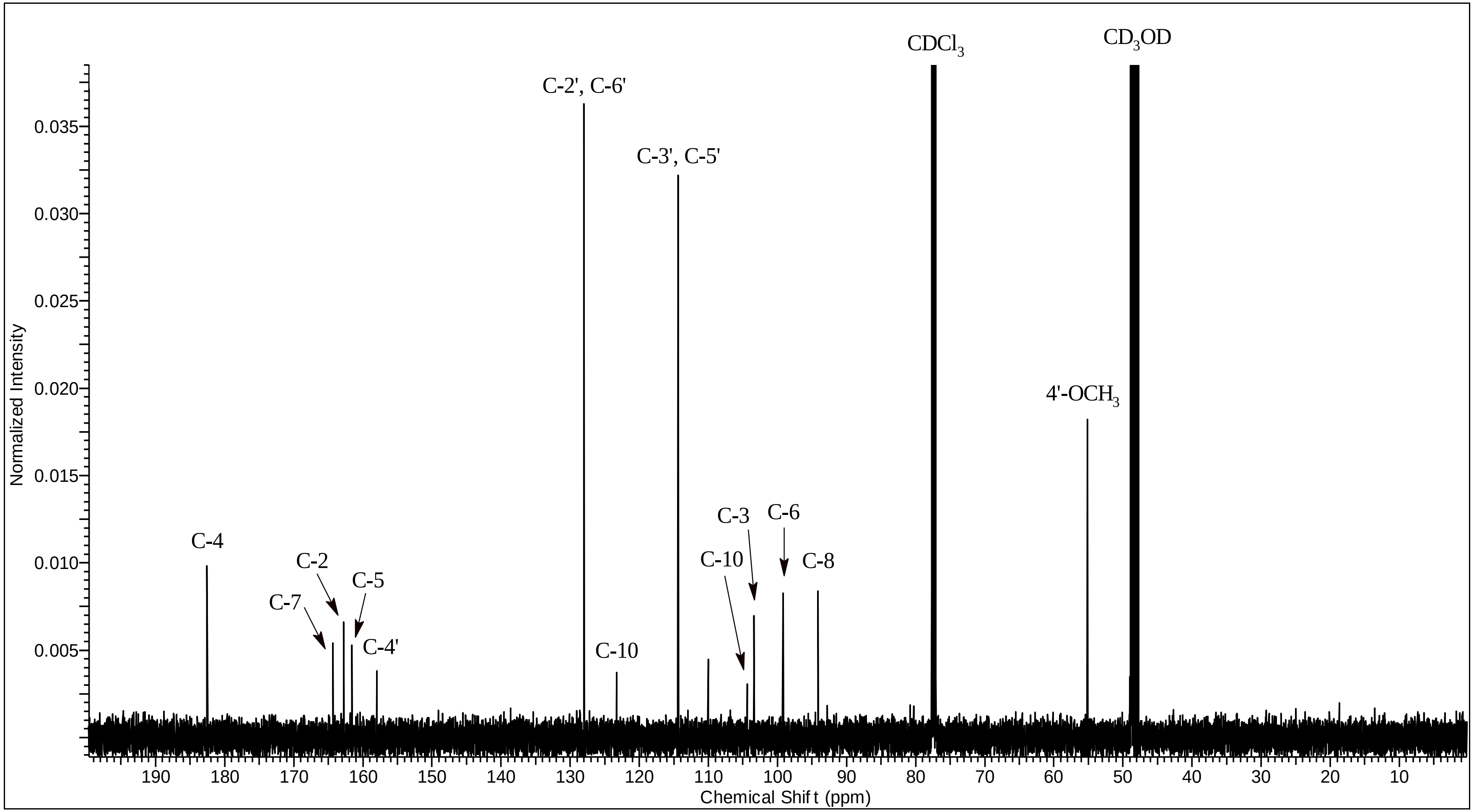

Figure 14. ^{1}H NMR spectrum of compound **8** (500 MHz in DMSO-*d*)

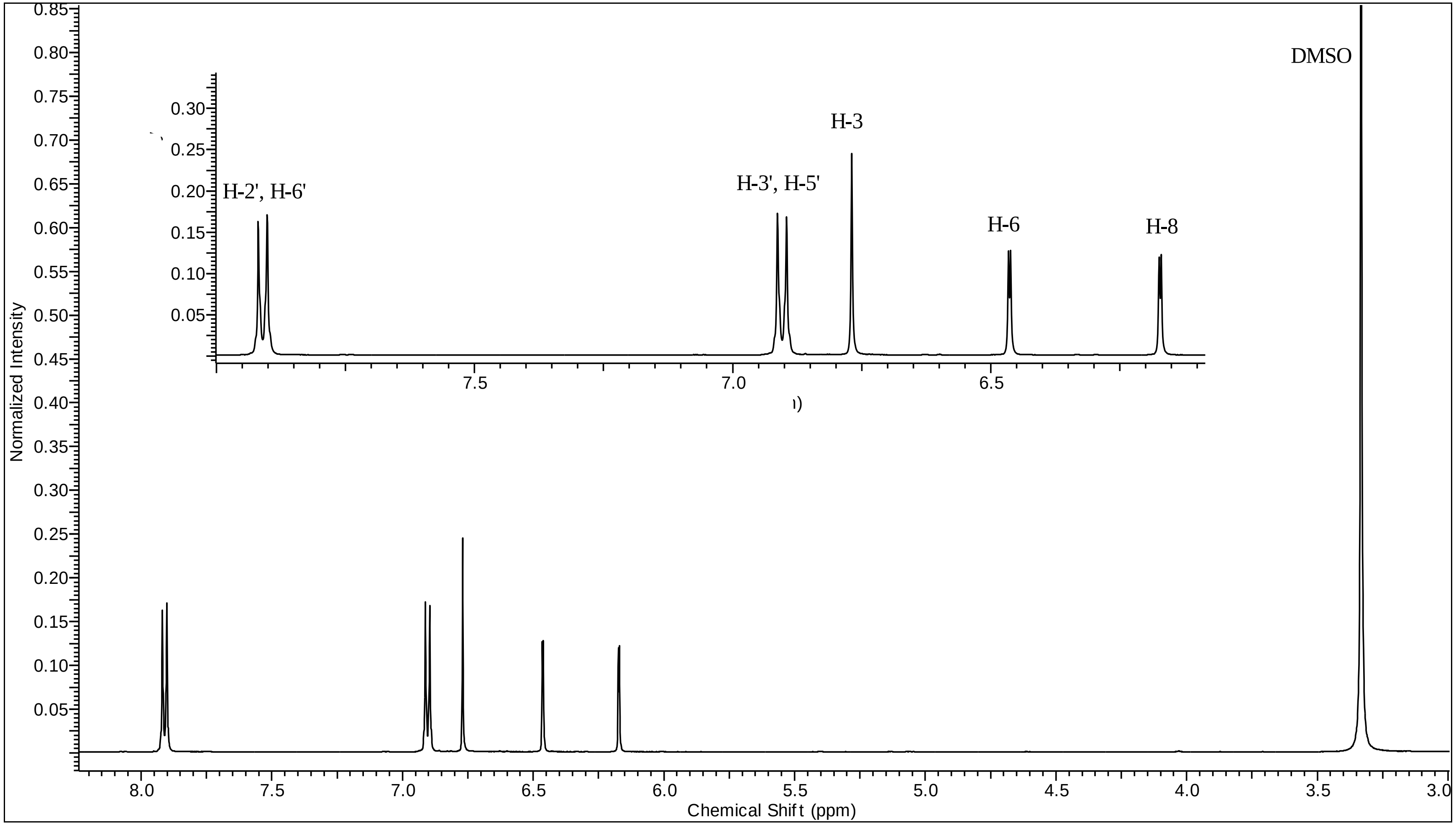

Figure 15. ^{13}C NMR spectrum of compound **8** (125 MHz in DMSO-*d*)

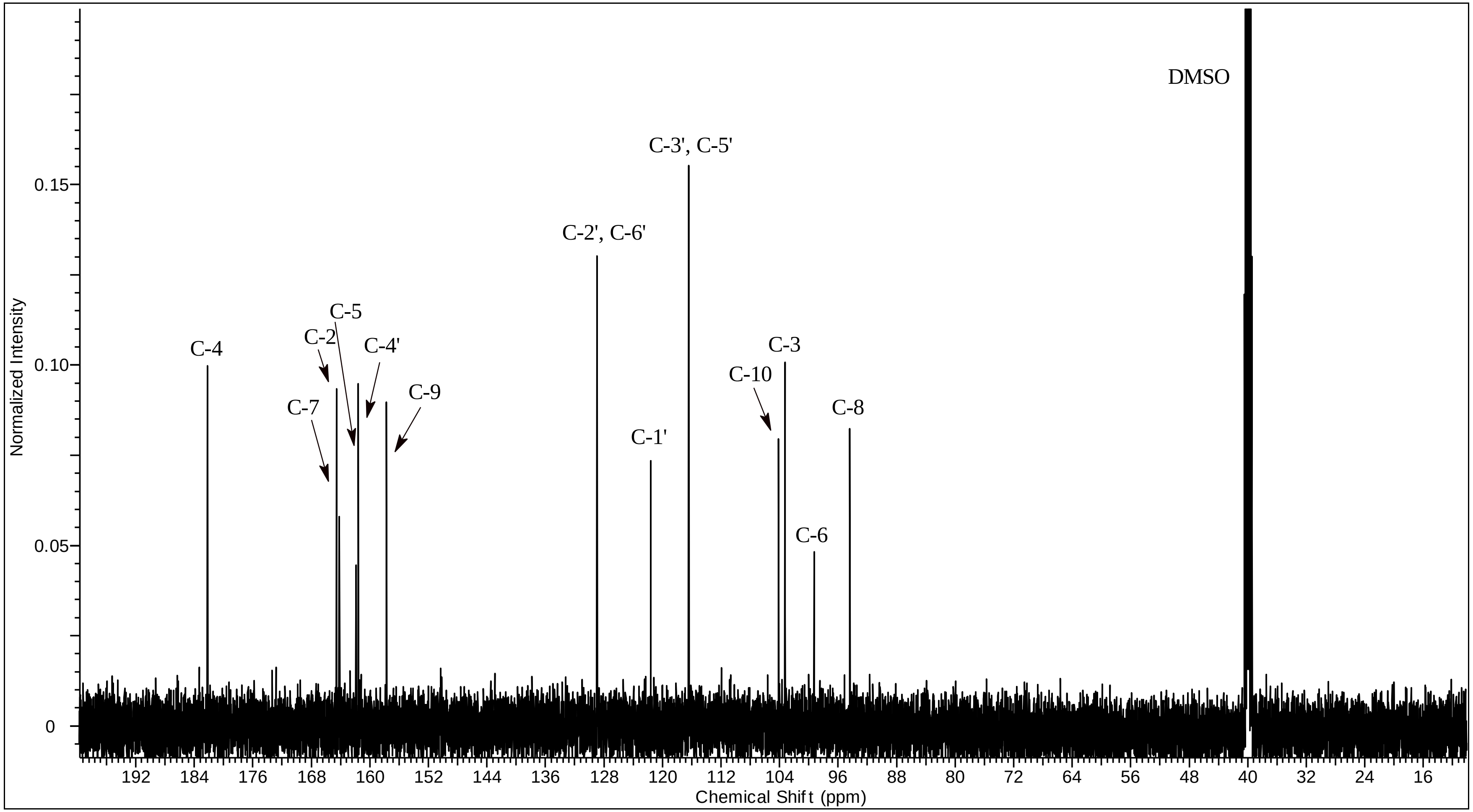

Figure 16. ^{1}H NMR spectrum of compound **9** (500 MHz in CD_3OD)

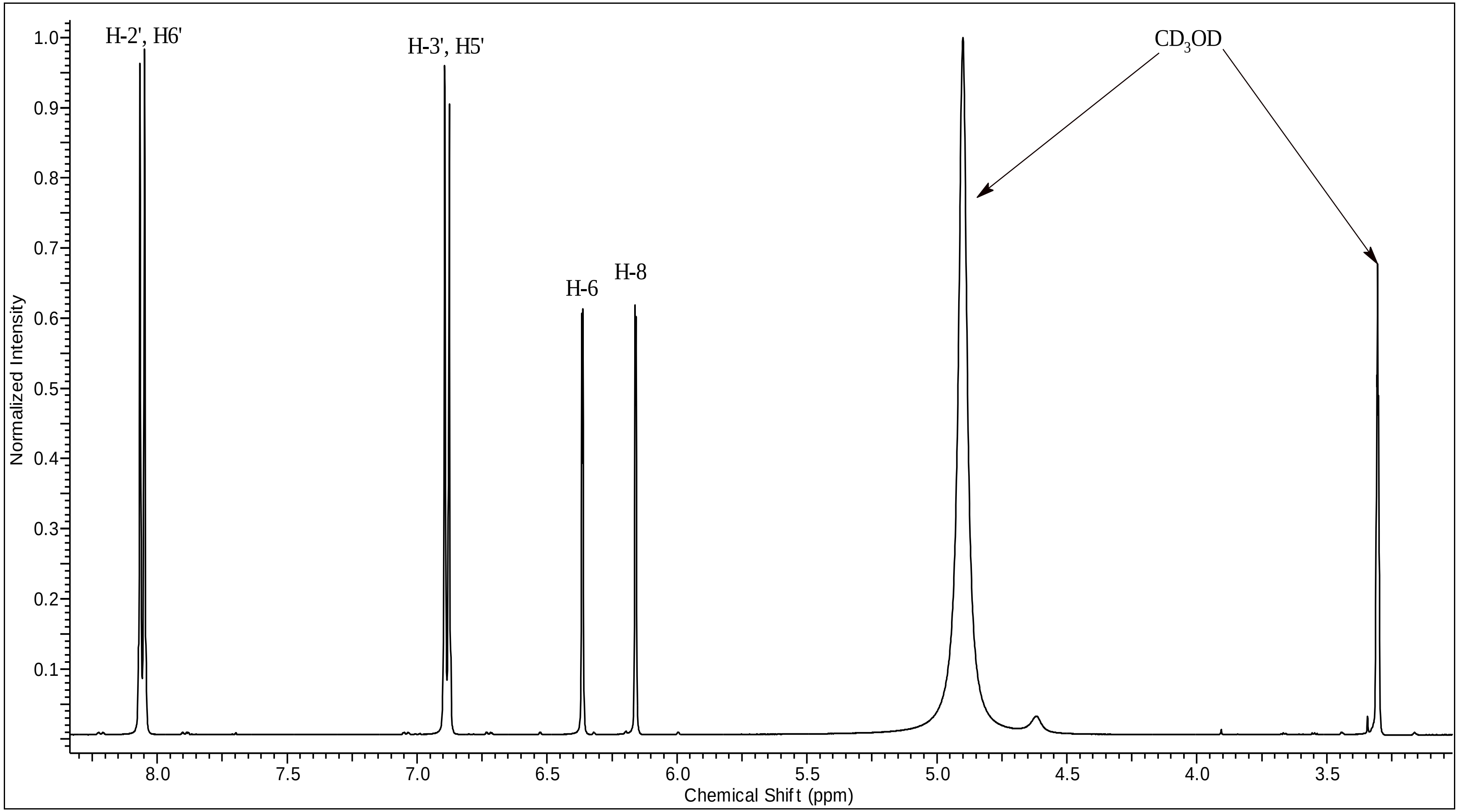

Figure 17. ^{13}C NMR spectrum of compound **9** (125 MHz in CD_3OD)

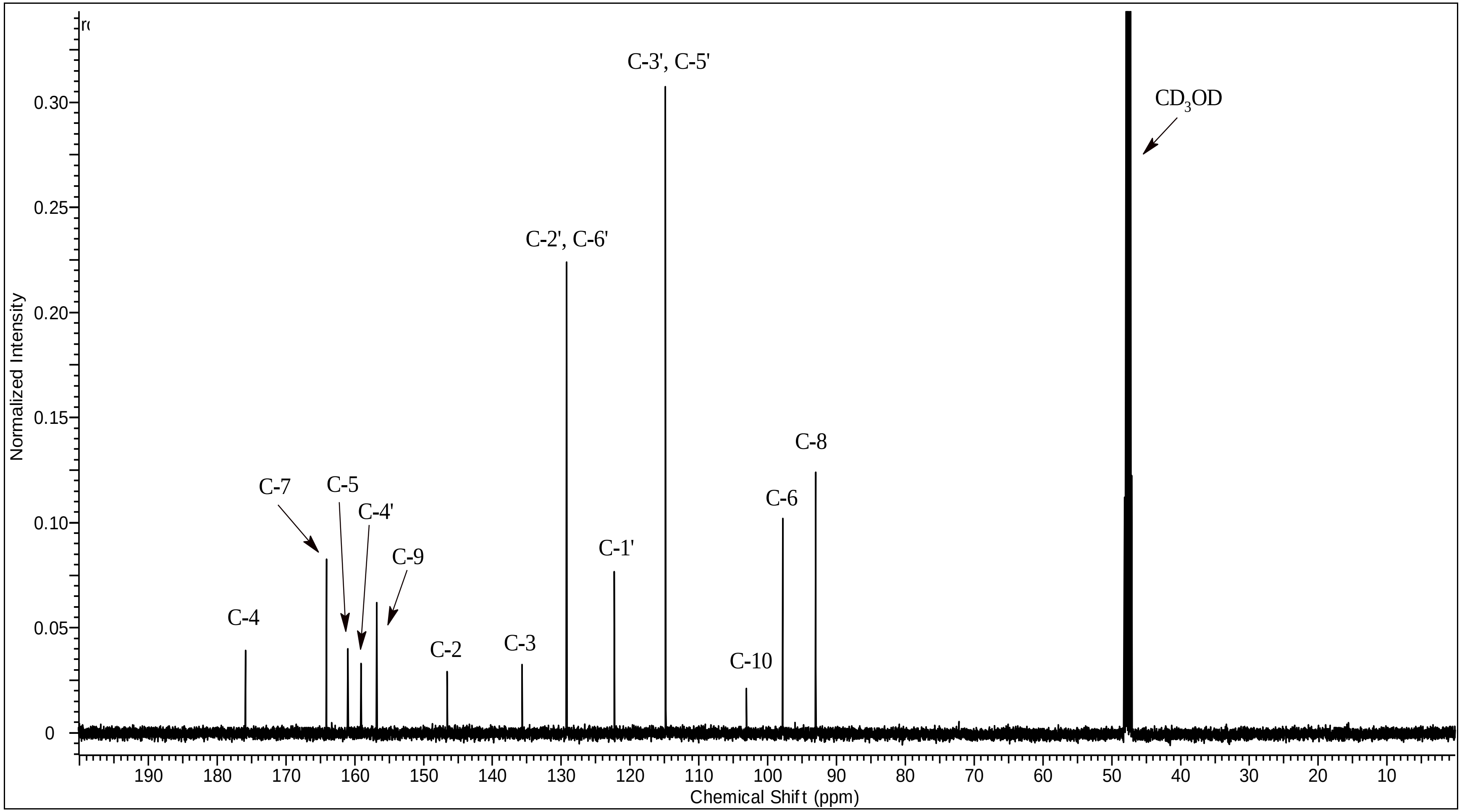

Figure 18. ^{1}H NMR spectrum of compound **10** (500 MHz in DMSO-*d*)

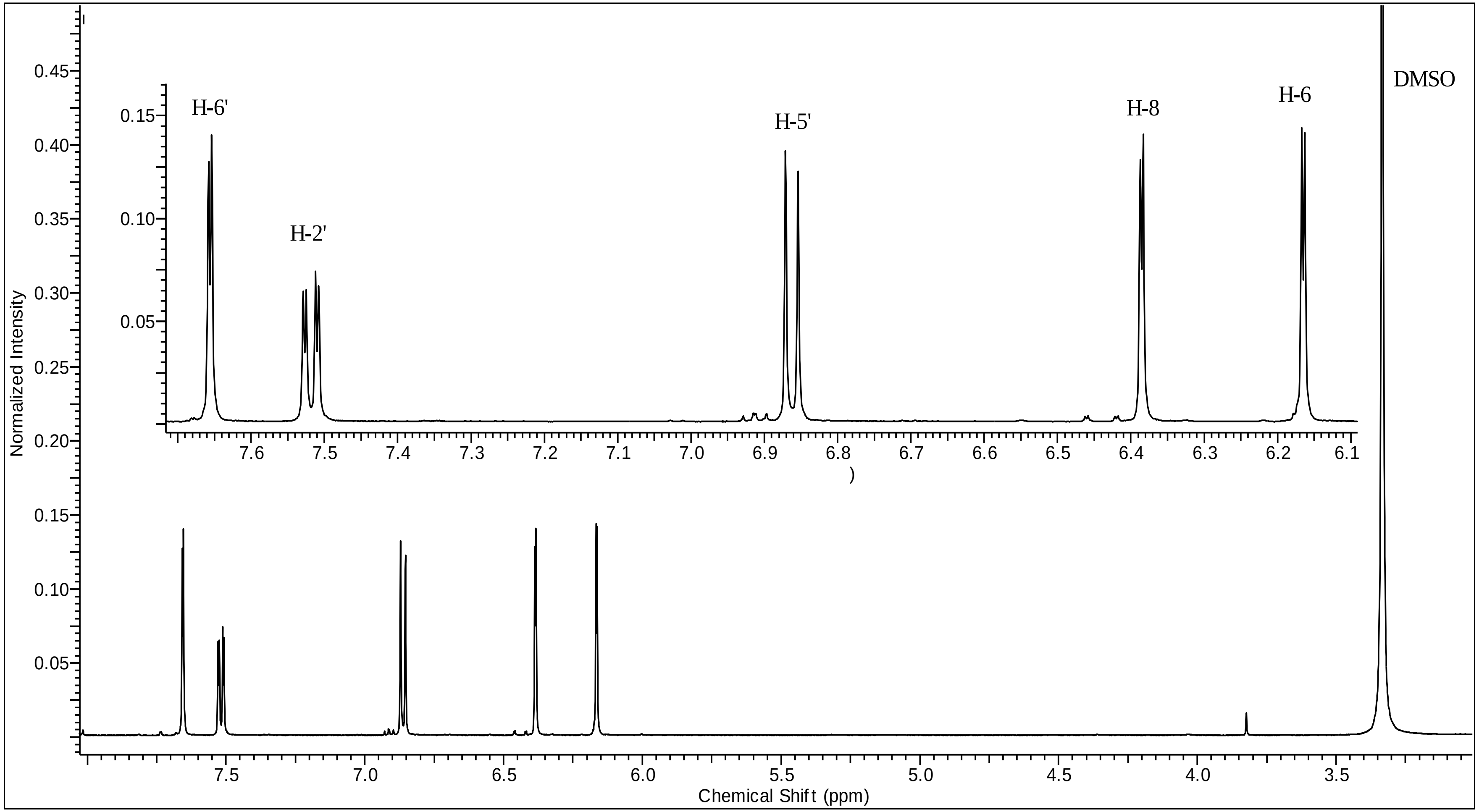

Figure 19. ^{13}C NMR spectrum of compound **10** (125 MHz in DMSO-*d*)

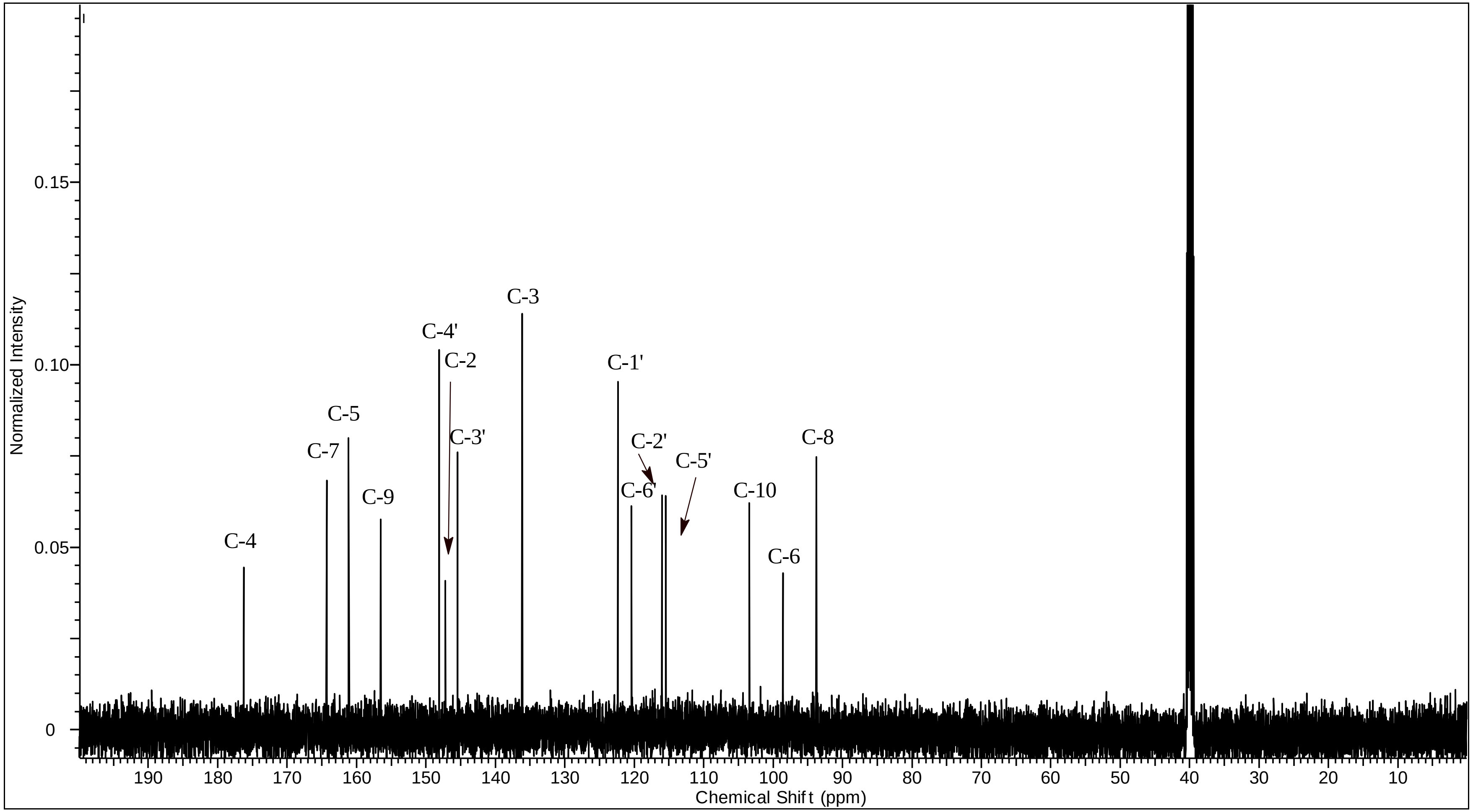

Figure 20. ^{1}H NMR spectrum of compound **11** (500 MHz in CD_3OD)

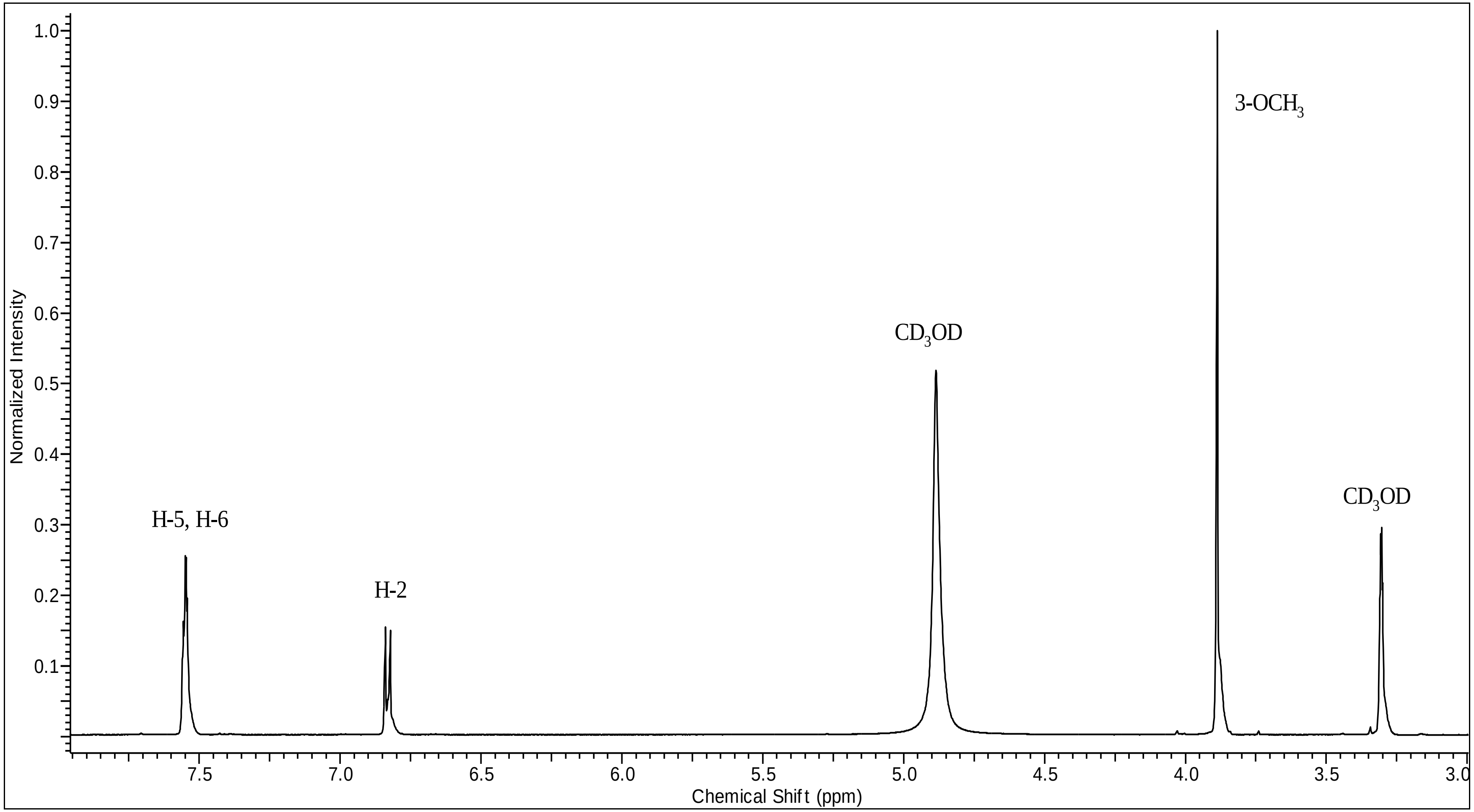

Figure 21. ^{13}C NMR spectrum of compound **11** (125 MHz in CD_3OD)

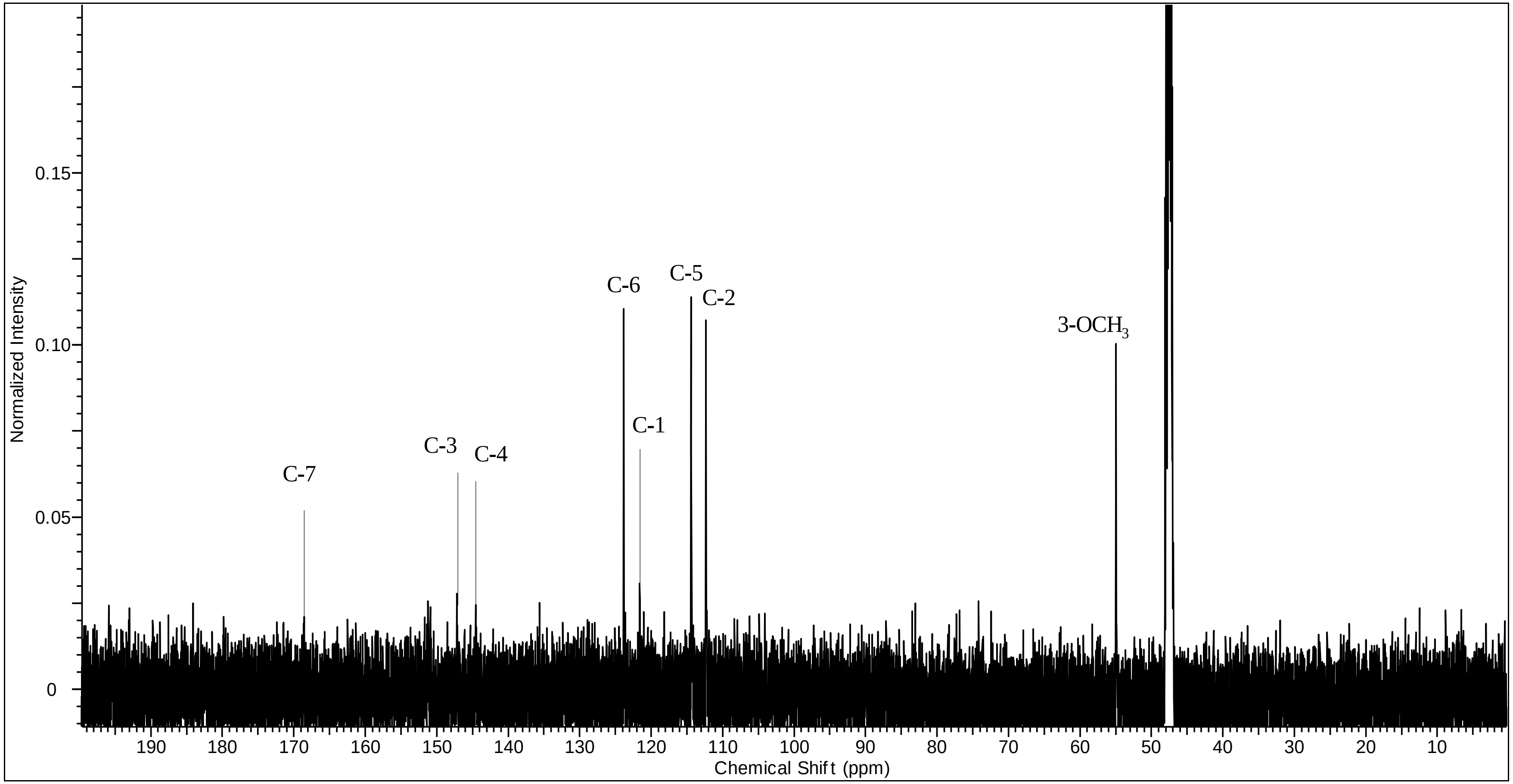

Supplement: Supplementary file 1 [file molecules-24-01469-s001.pdf]
